# Artificial Intelligence-Supported and App-Aided Cephalometric Analysis: Which One Can We Trust?

**DOI:** 10.3390/diagnostics15050559

**Published:** 2025-02-26

**Authors:** Senol Koz, Ozge Uslu-Akcam

**Affiliations:** 1 Private Practice, 34255 Istanbul, Turkey; kozsenol@hotmail.com; 2 Department of Orthodontics, Faculty of Dentistry, Ankara Yıldırım Beyazıt University, 06220 Ankara, Turkey

**Keywords:** artificial intelligence, cephalometric analysis, WebCeph, OneCeph, smartphone application

## Abstract

**Background**: This study aimed to compare the reproducibility and reliability of the AI-supported WebCeph and app-aided OneCeph cephalometric analysis programs with a manual analysis method and to evaluate the analysis times. **Methods**: The study material consisted of pretreatment lateral cephalograms from 110 cases. Cephalometric analyses were performed manually, using the WebCeph program, and using the OneCeph application. A total of 11 skeletal, 6 dental, and 3 soft tissue parameters were measured. Cephalometric analyses of 30 randomly selected cases were performed again using three methods. The analysis times were recorded. **Results**: The WebCeph program and OneCeph application are highly compatible with the manual analysis method in terms of all parameters, except for SN measurement. It was found that the WebCeph program and the OneCeph application demonstrated moderate agreement in U1-NA distance measurement, while statistically high agreement was observed among all three methods for other dental parameters. It was determined that there was a moderate agreement among the methods in terms of nasolabial angle, whereas a statistically high level of agreement was found for the other soft tissue parameters. The analysis time was found to be the lowest in the WebCeph program and the highest in the manual analysis method. **Conclusions**: The WebCeph program and OneCeph application showed a high degree of compatibility with the manual analysis method, except for SN, SNA, Gonial angle, Articular angle, U1-NA distance and nasolabial angle measurements. Due to the higher correlation between OneCeph and the manual method, it can be concluded that the OneCeph application is the best alternative to the manual method.

## 1. Introduction

The most important stage of an orthodontic treatment is the accurate diagnosis of malocclusion. The introduction of X-rays in orthodontic practice enabled the skeletal evaluation of malocclusion [1]. For many years, cephalometric analyses were conducted through manual tracings and measurements on radiographic printouts. Even though this method is considered to be the gold standard, it is a time-consuming and labor-intensive method that requires experience and skill, and it is also affected by factors such as the quality of the radiographs and human-related issues like fatigue, which has led researchers to alternative methods [2].

The digital revolution, which was experienced in the 2000s on a global level, also affected the field of dentistry and laid the foundation for the concept of digital orthodontics. Cephalometric analysis became faster and more accurate thanks to the development of software that allows drawing in digital media [3,4].

In orthodontics, programs developed by making use of artificial intelligence can perform cephalometric analysis by identifying anatomical reference points on lateral cephalometric radiographs through machine learning. The first study on this subject was carried out by Levy Mendel et al. in the 1980s, and subsequent studies have reported highly accurate results in comparison to traditional cephalometric analysis [5,6]. Advancing artificial intelligence applications aim to minimize physician errors and allow for analysis within minutes. Nowadays, many cephalometric analysis programs using artificial intelligence technology have been developed (WebCeph, AudaCeph, Carestream, CephX, Dental Imaging, OrthoDX, WeDoCeph, CS Imaging V8, etc.). One of the most popular of these programs is WebCeph (AssembleCircle Corp., Seongnam-si, Gyeonggi-do, Republic of Korea).

Smartphones offer many opportunities to humanity, and cephalometric analysis specially developed for these smartphones eliminated the need for a computer to conduct analysis. Thanks to these easily accessible programs that can be downloaded onto smartphones, cephalometric analysis can be performed in a very short period of time. Many app-aided cephalometric analysis programs have been developed (SmileCeph, CephNinja, SmartCeph Pro, OneCeph, etc.). One of these app-aided cephalometric programs is OneCeph, developed by Pavan Kumar Mamilapalli (Dr. M. Pavan Kumar, NXS Corp., Hyderabad, India). OneCeph is an application that can be downloaded to a mobile phone for free from the Google Play Store. Studies assessing the analysis conducted using the OneCeph software (https://play.google.com/store/apps/details?id=in.co.nxs.oneceph&hl=tr (accessed on 20 February 2025)) have reported the results to be consistent with manual analysis and other methods [7,8].

The speed, accuracy, and reproducibility of cephalometric analysis not only affect treatment planning, but also the outcomes of treatment, and offer remarkable convenience to the orthodontist. This study aimed to propose a reliable, free analysis to clinicians by comparing the reliability of one of the most popular AI-supported cephalometric analysis tool, WebCeph, and one of the most popular app-aided cephalometric analysis tools, OneCeph, and assessing them in terms of their analysis times. We aimed to determine the cephalometric analysis method that required the least amount of time and was reliable and reproducible for the orthodontist.

The null hypothesis was that there are no statistical differences among the cephalometric analysis methods regarding accuracy and analysis time.

## 2. Materials and Methods

All the procedures of the study were in accordance with the Declaration of Helsinki, and this retrospective study received approval from the Ethics Committee of Ankara Yıldırım Beyazıt University (date: 6 September 2022; approval no: 10).

The study’s sample size was calculated using G*Power software (version 3.1.9.213) developed by the Institute of Experimental Psychology at Heinrich Heine University in Dusseldorf, Germany. Based on the results of the power analysis calculations according to a previous data [9], a minimum sample size of 90 was determined to be required to achieve a test power (1-β) of 0.85. To prevent data loss, the determined sample size was increased by 20% to 110. No distinction was made between skeletal or dental malocclusions in the present study.

The lateral cephalometric radiographs used in the present study were obtained under standard conditions by using a Planmeca Promax 2D device (Planmeca OY Asentajankatu 6, 00880 Helsinki, Finland) in the patient’s natural head position, parallel to the midsagittal plane, and with X-rays perpendicular to the film. Cephalometric radiographs of 47 males and 63 females with a mean age of 14,6 years (between 10 and 32 years) were included in the study by considering the inclusion criteria.

The inclusion criteria were as follows:

* High-quality cephalograms that accurately demonstrated the cephalostat position without any artifacts that could obstruct the identification of anatomical sites.
* Patients in permanent dentition stage with cephalometric radiographs obtained prior to orthodontic treatment.
* Patients who did not have any significant deviations from normal.

The exclusion criteria were as follows:

* Cephalograms where the landmarks were not clearly defined.
* Cephalograms with significant double borders of the mandible.
* Individuals with craniofacial anomalies, asymmetries, or a history of craniofacial surgery.
* Individuals with significant dental abnormalities, diseases affecting cephalogram analysis, multiple missing teeth, or extensive crown-bridge restoration.

A total of 490 radiographs were reviewed, of which 400 met the eligibility criteria. The images were numbered, and 110 images were randomly selected as the study sample.

### 2.1. Study Design

#### 2.1.1. The Cephalometric Points Used in the Study

**Figure 1:**
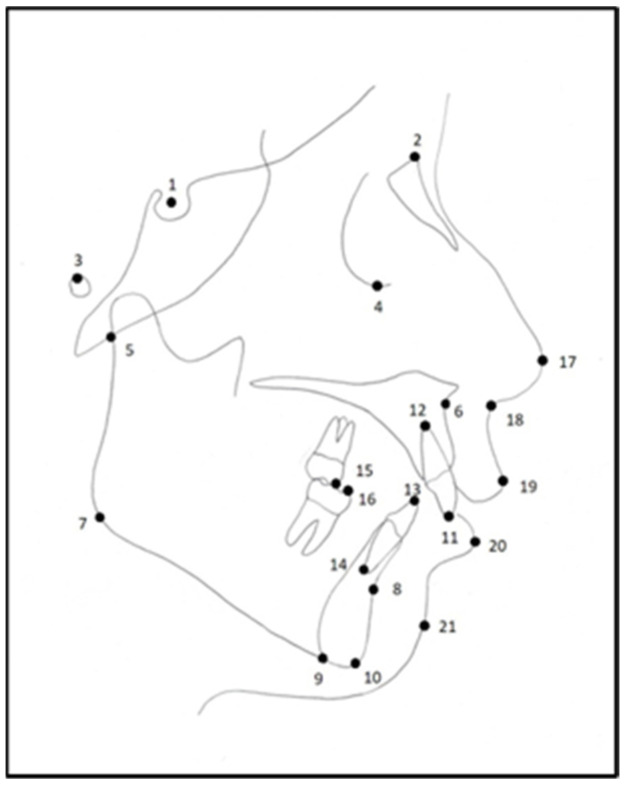
The cephalometric landmarks used in the study on a manual cephalometric tracing: 1—Sella; 2—Nasion; 3—Porion; 4—Orbita; 5—Articular; 6—Point A; 7—Gonion; 8—Point B; 9—Menton; 10—Gnathion; 11—upper incisor, U1 Incisal; 12—upper incisor, U1 Apex; 13—lower incisor, L1 Incisal; 14—lower incisor, L1 Apex; 15—upper 1st molar, U6 mesio-buccal tubercle; 16—lower 1st molar, L6 mesio-buccal tubercle; 17—Pronasale; 18—Subnasale; 19—Labium Superior; 20—Labium Inferior; 21—soft tissue pogonion point.

A total of 10 cephalometric skeletal landmarks, 6 dental landmarks, and 5 soft tissue landmarks were used (Figure 1).

#### 2.1.2. The Cephalometric Measurements Performed in the Study

Using these landmarks, 11 skeletal measurements, 6 dental measurements, and 3 soft tissue cephalometric measurements were performed (Table 1).

Before the measurements, the authors watched videos introducing the new analysis methods, and trial measurements were performed to minimize errors. The measurements were conducted by an author with 3 years of experience and were verified by the other author, with 20 years of experience, in order to eliminate experience-related errors. Measurements were limited to 5 analyses for each method per day, and a 30 min rest break was given between each analysis to minimize fatigue-caused errors.

#### 2.1.3. The Cephalometric Analysis Methods Used in the Study

1. Manual Method:

Translucent acetate paper (0.003 inches thick, 8″ × 10″ inches in size) specifically designed for orthodontic analysis was fixed to radiographic printouts. They were placed on a negatoscope in a darkened drawing room, and the anatomical points used in the present study were carefully marked using a Rotring drawing pen (0.3 mm) for both hard and soft tissue drawings. Angular and linear measurements were performed by the same authors using a protractor.

2. WebCeph Analysis Software

**Figure 2:**
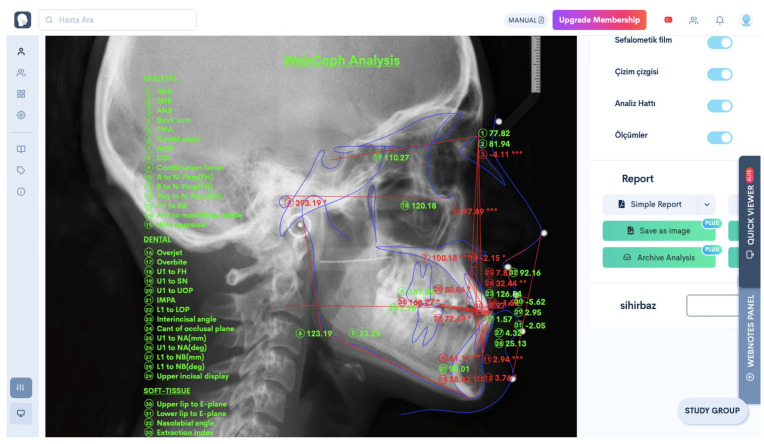
Analysis screen in WebCeph program. (Webceph program automatically adds *, ** or *** next to the cephalometric analysis results according to the amount of deviation from the norm values).

Digital radiographic images were saved in the ‘JPEG’ format on a MacBook Air 2017 computer (Apple Inc. One Apple Park Way, Cupertino, CA, USA). After signing up on the www.webceph.com website (https://webceph.com/tr/ (accessed on 15 September 2022)), the radiographic images were uploaded to the program in the ‘JPEG’ format by using an anonymous name. After the uploading process, the ‘Artificial Intelligence Digitization’ button was clicked to allow the program to automatically identify anatomical points. The points identified were then saved by clicking the ‘Save’ button. Then, an analysis list was opened by clicking the ‘Analysis’ tab, and measurements for the parameters to be used in the present study were performed (Figure 2) and then transferred to a table prepared in the Microsoft Excel software (One Microsoft Way, Washington, DC, USA).

3. OneCeph Analysis Application

**Figure 3:**
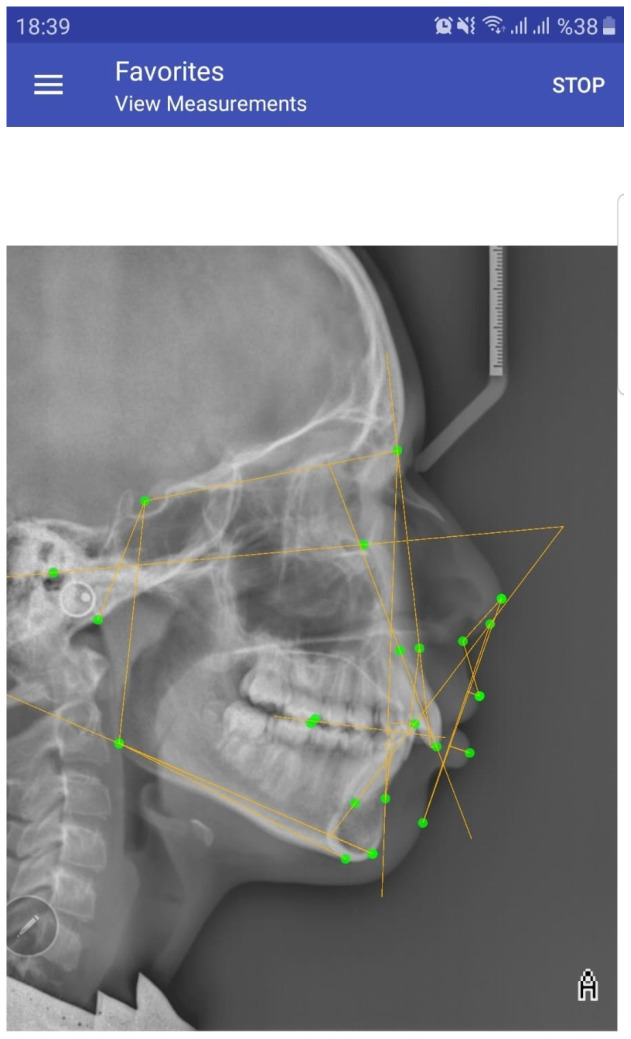
Analysis screen in OneCeph application.

Digital radiographic images were saved in the ‘JPEG’ format on a Samsung Galaxy S20 FE smartphone (Samsung, Yeongtong Suwon, Korea) with a screen size of 5.7 inches and a resolution of 1250 × 1592 pixels. The OneCeph application was downloaded free of charge to the relevant phone from the Google Play Store. After opening the program, a radiographic image saved in the phone’s gallery was selected and saved in the program under an anonymous name. After the calibration, measurements for the parameters to be used in the present study were sequentially performed (Figure 3) and the results were transferred to a table prepared in the Microsoft Excel software (Version 2411).

### 2.2. Statistical Analysis

The data were analyzed using the SPSS 21 (SPSS Inc., Chicago, IL, USA; version 15.0 for Windows) package program. The compatibility between cephalometric analysis methods was evaluated by using the ICC (Inter-Class Correlation) method. Intra-observer reliability was evaluated for 30 randomly selected cases by using the ICC (Intra-Class Correlation) method. The Kruskal–Wallis H Test was used for comparisons between the duration of the analysis methods. A significance level was set at 0.05; it was stated that significant agreement existed if *p* < 0.05, whereas there was no significant agreement if *p* > 0.05.

### 2.3. Method Error

To determine the intra-observer reliability, 30 randomly selected radiographs were reevaluated for cephalometric measurements by the same researchers after 1 month by using all three methods. During measurements, a digital chronometer was used for each measurement method; the total analysis time was calculated, and the results were transferred to the relevant Excel table.

## 3. Results

### 3.1. Intraobserver Reliability

The ICC values used to indicate different levels of intra-observer reliability were set as poor reliability (ICC < 0.50), moderate reliability (0.50 ≤ ICC < 0.75), good reliability (0.75 ≤ ICC < 0.90), and excellent reliability (ICC ≥ 0.90). Considering the intra-observer reliability in the present study, a high degree of correlation was observed in the repeated measurements for all three cephalometric analysis methods. The lowest correlation was found in FMA angle measurements (ICC = 0.784) for the repeated measurements of manual analysis, whereas there was a high level of correlation for the other parameters in all the analysis methods (ICC > 0.85).

### 3.2. Skeletal, Dental, and Soft Tissue Parameters

There was a statistically low level of agreement in the SN measurement between the WebCeph program and the manual analysis method (ICC = 0.215) and between the WebCeph program and the OneCeph application (ICC = 0.175). However, there was a moderate agreement between the manual analysis method and the WebCeph program for the SNA, Gonial, and Articular angle measurements. Statistically high levels of agreement were found for the other skeletal parameters (Table 2).

It was found that the WebCeph program and the OneCeph application demonstrated moderate agreement in U1-NA distance measurement, while statistically high agreement was observed among all three cephalometric analysis methods for other dental parameters (Table 3).

It was determined that there was a moderate agreement among the three cephalometric analysis methods in terms of nasolabial angle measurement, whereas a statistically high level of agreement was found for other soft tissue parameters (Table 4).

### 3.3. Duration of the Analysis

The cephalometric analysis of 30 randomly selected radiographs was performed by using all three analysis methods, and the analysis times were recorded by using a digital chronometer. The mean cephalometric analysis times were calculated for all three cephalometric analysis methods. The shortest analysis time was obtained with the WebCeph program (1.25 min), whereas the longest one was obtained using the manual analysis method (9.10 min) (Table 5).

Since the data of the analysis times had a non-normal distribution, the Kruskal–Wallis H test was used for comparisons between the three groups, and statistically significant differences were observed in terms of the durations (*p* < 0.05) (Table 5). The analysis times obtained with the AI-supported WebCeph program were significantly lower when compared to the manual analysis method and the OneCeph application. The analysis time obtained using the OneCeph application was significantly lower in comparison to the analysis time obtained with the manual analysis method (Table 5).

## 4. Discussion

The size of the material used in a study is a factor that affects the reliability. Studies carried out on reliability emphasize the subject of sample size [10]. In the present study, a relatively high sample size was used when compared to previous studies. The measurements were conducted by an author with 3 years of experience and were verified by another author with 20 years of experience in order to eliminate experience-related errors. Furthermore, the measurements were performed in standard conditions and limited numbers.

The most important criteria for the acceptability and reliability of cephalometric analysis are the high accuracy and reproducibility of the analysis [9]. Therefore, in this study, the accuracy and reproducibility of the AI-supported WebCeph program and the app-aided OneCeph application were compared to the gold standard manual analysis method.

It has been reported in previous studies that inter-observer error is higher than intra-observer error, and this error is related to the rater’s experience [11,12,13,14]. Due to high inter-observer error rates, measurements were made by the same observers. In this study, after the first measurements, 30 cephalometric radiographs were randomly selected and re-evaluated by the same researchers with three different methods, and a high intra-observer correlation was found. Similarly, a high intra-observer correlation coefficient was reported in a previous study carried out by Sayınsu et al. [15]. Zamrik and İseri emphasized that all parameters were statistically significantly reproducible [8].

The determination of the anatomical reference points is the most important factor in the accuracy of the analysis [16]. In the AI-supported WebCeph analysis program, unlike the other two methods, anatomical reference points are determined by artificial intelligence, and then corrections can be made by the operator, and the analysis is conducted automatically [17]. In a study comparing two different AI-supported analysis programs with manual analysis, Duran et al. recommended operator intervention to increase the accuracy of the analysis [18]. Çoban et al. compared the WebCeph program to the Dolphin computerized analysis program and found statistically significant differences in the SNB, U1-NA, interincisal angle, and Li-E line measurements [19]. In this study, the measurement of SN length and nasolabial angle yielded the lowest level of agreement with the other two measurement methods (Table 2 and Table 4). In previous studies carried out on this subject, the error in SN measurement was attributed to the insufficient visibility of the nasofrontal suture, while the error in nasolabial angle measurement was related to the difficulty in detecting anatomical points in tissues with rounded curvatures [20,21]. Therefore, it is considered that the WebCeph program might be inadequate in detecting anatomical points in areas with rounded curvatures and insufficient visibility. Hence, as recommended by many other researchers, it can be recommended for the operator to make corrections after the program determines the anatomical reference points [14,22,23].

The OneCeph application, which can be downloaded for free on smartphones, eliminates the need to have a computer for cephalometric analysis, and it provides healthcare professionals with the opportunity to perform cephalometric analysis quickly and free of charge, although there are relatively few studies carried out on this new application. In a study where Shettigar et al. compared the OneCeph application with the Dolphin computerized analysis program, a statistically low agreement was found for the SNB and IMPA measurements, as well as the FMA angle measurements [24]. In a study carried out by Shresta and Kandel, the OneCeph application was compared to manual cephalometric analysis, and the measurements were found to be consistent, except for the L1-NB linear measurement [25]. Barbhuiya et al. compared the manual method to the OneCeph application and found consistent measurements for all parameters [7]. In our study, there was generally high agreement between the OneCeph application and the manual analysis method, but partially lower agreement was found for the nasolabial angle measurements (Table 4). Similarly, in a study carried out by Zamrik and İşeri comparing the manual analysis method and the OneCeph application, a statistically significant difference was found in the nasolabial angle measurement. The authors attributed this difference to the impractical technique of determining the nasal tangent line in the OneCeph application [8]. In the present study, it was observed that artificial intelligence is still insufficient for detecting anatomical reference points in curved areas and areas with unclear visibility. According to the findings of this study, the null hypothesis that all three methods were no different can be rejected regarding the measurements of SN, SNA, Gonial angle, Articular angle, U1-NA distance, and nasolabial angle. The null hypothesis can be accepted for the other skeletal, dental, and soft tissue parameters.

In their studies comparing AI-supported and app-aided cephalometric analyses by using the manual analysis method, Meriç and Naoumova found angular measurements to be more consistent in comparison to dimensional measurements [9]. In similar studies carried out on the same subject, researchers also found dimensional measurements to be less consistent and claimed that this may be due to image distortion and calibration issues [26,27,28].

Due to its many appealing features that might make orthodontic treatment planning and patient record gathering easier, WebCeph is an AI-supported orthodontic and orthognathic online platform that has been gaining popularity recently. These features consist of picture archiving, automatic superimposition, visual treatment simulation, automated tracing, and analysis of the cephalometric structure. In addition to these advantages, AI also needs large amounts of data consisting of cephalometric images. AI-based digital software requires high-resolution lateral cephalograms and the absence of structure superimposition because of possible interferences with the algorithm for landmark identification.

The opportunity to perform cephalometric analysis quickly and accurately provides significant convenience to healthcare professionals during the diagnosis. In a study carried out by Chen et al., a remarkable difference was reported between experienced and inexperienced users in drawing anatomical structures and placing points. Furthermore, their research revealed that the level of experience among the methods would affect the analysis times [29].

In the present study, the shortest analysis time was achieved with the WebCeph program (1 min 24 s), whereas the average analysis time in the OneCeph application was 1 min longer (2 min 14 s). The highest time was obtained in the traditional manual analysis method (9 min 11 s) (Table 5). In a study carried out by Shresta et al., the time for manual analysis was measured to be 15 min and 30 s, whereas the time for the OneCeph application was found to be 4 min and 50 s [20]. In a comparative study on manual analysis and the smartphone application CephNinja, Sayar et al. found an average time of 4 min and 32 s for the manual analysis method and 2 min and 46 s for the CephNinja application [30]. The longer time required for the manual analysis method in our study, compared to other methods, is consistent with the results reported in previous studies in terms of the shorter times in AI-supported and app-aided analysis methods [31,32].

This study has some limitations. Although a larger number of subjects and parameters were assessed in this study compared to previous studies, future studies encompassing a greater number of subjects and cephalometric parameters are suggested. Increasing the number of subjects and parameters can enable the assessment of the reliability of the methods with greater accuracy. Additionally, it would be better to evaluate analysis times in a larger number of cases. Including various cases of malocclusion in the inclusion criteria would provide a more comprehensive evaluation of the methods being studied.

## 5. Conclusions

Based on the findings presented, the following conclusions can be drawn:

* All three cephalometric analysis methods were found to have a high degree of reproducibility.
* The manual analysis method was found to be very highly compatible with the app-aided OneCeph cephalometric analysis program and highly compatible with the AI-supported WebCeph cephalometric analysis program, except for the SN, SNA, Gonial angle, Articular angle, U1-NA distance, and nasolabial angle measurements.
* Both analysis methods can be reliably used as alternatives to the manual analysis method.
* It was determined that the AI-supported WebCeph cephalometric analysis program was the fastest analysis program when compared to the other two analysis methods.
* The app-aided OneCeph cephalometric analysis application is much more compatible with the ‘gold standard’ manual analysis method and allows for quicker analysis.

## Figures and Tables

**Table 1:** The cephalometric measurements used in this study.

**Skeletal Measurements**	
S-N (mm)	Distance between the Sella point and Nasion point
SNA (°)	Angle between the Sella–Nasion line and the Nasion–Point A line
SNB (°)	Angle between the Sella–Nasion line and the Nasion–Point B line
ANB (°)	Angle between the Nasion–A and Nasion–B lines
WITTS (mm)	Distance between the perpendiculars drawn from Points A and B to the occlusal plane
GOGN-SN (°)	Angle between the Sella–Nasion line and the Gonion–Gnation line
FMA (°)	Angle between the Porion–Orbitale line and the Gonion–Menton line
SADDLE (°)	Angle between the Sella, Nasion, and Articular points
GONIAL (°)	Angle between the Articular, Gonion, and Menton points
ARTICULAR (°)	Angle between the Sella, Articular, and Gonion points
SUM (°)	Sum of the Saddle angle, Gonial angle, and Articular angle
**Dental Measurements**	
U1-NA ANGLE (°)	Angle between the long axis of the upper first incisor tooth and the N–A line
U1-NA DISTANCE (mm)	Sagittal distance from the most anterior point of the crown of the upper first incisor tooth to the N–A line
L1-NB ANGLE (°)	Angle between the long axis of the lower first incisor tooth and the N–B line
L1-NB DISTANCE (mm)	Sagittal distance from the most anterior point of the crown of the lower first incisor tooth to the N–B line
IMPA (°)	Angle between the long axis of the lower first incisor and the mandibular plane
INTERINCISAL ANGLE (°)	Angle between the long axis of the upper first incisor tooth and the long axis of the lower first incisor tooth
**Soft Tissue Measurements**	
LS-E (mm)	Distance from the Labium Superior point to Plane E
LI-E (mm)	Distance from the Labium Inferior point to Plane E
NASOLABIAL ANGLE (°)	Angle between the Pronasale, Subnasale, and Labium Superior points

**Table 2:** The correlation coefficients between the methods for the skeletal parameters (MAN: manual cephalometric analysis; WEB: WebCeph cephalometric analysis program; ONE: OneCeph application).

Skeletal Parameters		Correlation Coefficients Between the Methods				
			Correlation Between the Methods	Confidence Interval of 95%		
				Lower Threshold	Upper Threshold	*p*
1	SN-MAN (mm)	SN MAN-SN WEB	0.215	−0.137	0.473	0.005
2	SN-WEB (mm)	SN MAN-SN ONE	0.939	0.911	0.958	0.000
3	SN-ONE (mm)	SN WEB-SN ONE	0.175	−0.125	0.413	0.021
1	SNA-MAN (°)	SNA MAN-SNA WEB	0.692	0.390	0.826	0.000
2	SNA-WEB (°)	SNA MAN-SNA ONE	0.875	0.808	0.917	0.000
3	SNA-ONE (°)	SNA WEB-SNA ONE	0.798	0.681	0.869	0.000
1	SNB-MAN (°)	SNB MAN-SNB WEB	0.883	0.829	0.920	0.000
2	SNB-WEB (°)	SNB MAN-SNB ONE	0.912	0.862	0.943	0.000
3	SNB-ONE (°)	SNB WEB-SNB ONE	0.911	0.870	0.939	0.000
1	ANB-MAN (°)	ANB MAN-ANB WEB	0.845	0.434	0.934	0.000
2	ANB-WEB (°)	ANB MAN-ANB ONE	0.944	0.919	0.962	0.000
3	ANB-ONE (°)	ANB WEB-ANB ONE	0.873	0.482	0.948	0.000
1	Witts-MAN (mm)	Witts MAN–Witts WEB	0.906	0.863	0.936	0.000
2	Witts-WEB (mm)	Witts MAN–Witts ONE	0.892	0.821	0.932	0.000
3	Witts-ONE (mm)	Witts WEB–Witts ONE	0.915	0.781	0.957	0.000
1	GoGn/SN-MAN (°)	GoGn/SN MAN-GoGn/SN WEB	0.953	0.931	0.968	0.000
2	GoGn/SN-WEB (°)	GoGn/SN MAN-GoGn/SN ONE	0.963	0.946	0.975	0.000
3	GoGn/SN-ONE (°)	GoGn/SN WEB-GoGn/SN ONE	0.952	0.917	0.970	0.000
1	FMA-MAN (°)	FMA MAN-FMA WEB	0.933	0.700	0.973	0.000
2	FMA-WEB (°)	FMA MAN-FMA ONE	0.827	0.263	0.932	0.000
3	FMA-ONE (°)	FMA WEB-FMA ONE	0.891	0.805	0.934	0.000
1	Saddle-MAN (°)	Saddle MAN–Saddle WEB	0.849	0.468	0.935	0.000
2	Saddle-WEB (°)	Saddle MAN–Saddle ONE	0.866	0.358	0.949	0.000
3	Saddle-ONE (°)	Saddle WEB–Saddle ONE	0.928	0.895	0.950	0.000
1	Gonial-MAN (°)	GoniaL-MAN–Gonial WEB	0.706	−0.188	0.898	0.000
2	Gonial-WEB (°)	Gonial-MAN–Gonial ONE	0.916	0.867	0.945	0.000
3	Gonial-ONE (°)	Gonial-WEB–Gonial ONE	0.78	−0.135	0.927	0.000
1	Articular-MAN (°)	Articular MAN–Articular WEB	0.732	−0.129	0.904	0.000
2	Articular-WEB (°)	Articular MAN–Articular ONE	0.892	0.623	0.953	0.000
3	Articular-ONE (°)	Articular WEB–Articular ONE	0.825	0.558	0.913	0.000
1	SUM-MAN (°)	SUM MAN-SUM WEB	0.890	0.840	0.925	0.000
2	SUM-WEB (°)	SUM MAN-SUM ONE	0.883	0.845	0.927	0.000
3	SUM-ONE (°)	SUM WEB-SUM ONE	0.943	0.858	0.971	0.000

**Table 3:** Correlation coefficients between the methods for the dental parameters (MAN: manual cephalometric analysis; WEB: WebCeph cephalometric analysis program; ONE: OneCeph application).

Dental Parameters		Correlation Coefficients Between the Methods				
			ICC	Confidence Interval of 95%		
				Lower Threshold	Upper Threshold	*p*
1	U1/NA-MAN (°)	U1/NA MAN-U1/NA WEB	0.89	0.673	0.949	0.000
2	U1/NA-WEB (°)	U1/NA MAN-U1/NA ONE	0.94	0.790	0.973	0.000
3	U1/NA-ONE (°)	U1/NA WEB-U1/NA ONE	0.911	0.869	0.939	0.000
1	U1-NA-MAN (mm)	U1-NA MAN-U1-NA WEB	0.767	0.576	0.861	0.000
2	U1-NA-WEB (mm)	U1-NA MAN-U1-NA ONE	0.901	0.856	0.932	0.000
3	U1-NA-ONE (mm)	U1-NA WEB-U1-NA ONE	0.745	0.589	0.837	0.000
1	L1/NB-MAN (°)	L1/NB MAN-L1/NB WEB	0.862	0.527	0.940	0.000
2	L1/NB-WEB (°)	L1/NB MAN-L1/NB ONE	0.948	0.809	0.978	0.000
3	L1/NB-ONE (°)	L1/NB WEB-L1/NB ONE	0.91	0.862	0.940	0.000
1	L1-NB-MAN (mm)	L1-NB MAN-L1-NB WEB	0.947	0.921	0.964	0.000
2	L1-NB-WEB (mm)	L1-NB MAN-L1-NB ONE	0.96	0.942	0.973	0.000
3	L1-NB-ONE (mm)	L1-NB WEB-L1-NB ONE	0.959	0.929	0.974	0.000
1	IMPA-MAN (°)	IMPA MAN-IMPA WEB	0.902	0.767	0.949	0.000
2	IMPA-WEB (°)	IMPA MAN-IMPA ONE	0.937	0.896	0.960	0.000
3	IMPA-ONE (°)	IMPA WEB-IMPA ONE	0.833	0.508	0.923	0.000
1	Interincisal-MAN (°)	Interincisal MAN–Interinc WEB	0.914	0.392	0.971	0.000
2	Interincisal-WEB (°)	Interincisal MAN–Interinc ONE	0.943	0.579	0.980	0.000
3	Interincisal-ONE (°)	Interincisal WEB–Interinc ONE	0.955	0.934	0.969	0.000

**Table 4:** Correlation coefficients between the methods for the soft tissue parameters (MAN: manual method; WEB: WebCeph program; ONE: OneCeph application).

Soft Tissue Parameters		Correlation Coefficients				
			ICC	Confidence Interval of 95%		
				Lower Threshold	Upper Threshold	*p*
1	Ls-E MAN (mm)	Ls-E MAN-LS-E WEB	0.93	0.898	0.952	0.000
2	Ls-E WEB (mm)	Ls-E MAN-LS-E ONE	0.949	0.917	0.967	0.000
3	Ls-E ONE (mm)	Ls-E WEB-LS-E ONE	0.955	0.908	0.975	0.000
1	Li-E MAN (mm)	Li-E MAN-Li-E WEB	0.937	0.872	0.964	0.000
2	Li-E WEB (mm)	Li-E MAN-Li-E ONE	0.964	0.937	0.978	0.000
3	Li-E ONE (mm)	Li-E WEB-Li-E ONE	0.966	0.949	0.977	0.000
1	NL-MAN (°)	NL MAN-NL WEB	0.588	−0.099	0.813	0.000
2	NL-WEB (°)	NL MAN-NL ONE	0.678	0.530	0.780	0.000
3	NL-ONE (°)	NL WEB-NL ONE	0.505	−0.177	0.769	0.000

**Table 5:** Statistical evaluation of duration of analysis methods.

		Group						Kruskal–Wallis H Test		
		*n*	Mean	Median	Min	Max	Standard Deviation	H	*p*	Paired Comparison
Time (min)	1 = MANUAL	30	9.10	9.11	8.55	9.38	0.17	79.419	0.0001	2–1
	2 = WEBCEPH	30	1.25	1.24	1.23	1.35	0.03			2–3
	3 = ONECEPH	30	2.14	2.14	2.10	2.20	0.02			3–1
	TOTAL	90	4.16	2.14	1.23	9.38	3.53			

## Data Availability

The data presented in this study are available upon request from the corresponding authors.
